# Vigorous physical activity and weight status in school-aged children: a cross-sectional study

**DOI:** 10.3389/fpubh.2024.1402780

**Published:** 2024-06-12

**Authors:** Weijun Yu, Shuanglu Xu, Xiaojie Dai, Huiying Fan

**Affiliations:** ^1^Faculty of Sport, College of Teacher Education, Taizhou University, Taizhou, China; ^2^School of Physical Education, Shanghai Normal University, Shanghai, China

**Keywords:** vigorous physical activity, weight status, school-aged children, cross-sectional study, association

## Abstract

**Aim:**

To explore the association between VPA and weight status in adolescents.

**Methods:**

The 2017/2018 Health Behavior in School-aged Children survey (HBSC) targeted children and adolescents aged 11, 13 and 15. A systematic multistage stratified cluster randomized sampling method was used in each participating country. The 2017/2018 survey enrolled over 240,951 adolescents across 45 countries and regions. Frequency of VPA, weight status and confounding factors were collected using a self-reported questionnaire.

**Results:**

Compared to daily VPA, less frequent VPA was linked to higher odds of obesity. For example, those who participating in VPA for 4–6 times a week (OR = 1.10, 95% CI = 1.06–1.13), 2–3 times a week (OR = 1.21, 95% CI = 1.17–1.25), or once a week (OR = 1.21, 95% CI = 1.16–1.25) all have higher odds of abnormal weight status. For boys, the frequency of 4–6 times a week (OR = 1.09, 95% CI = 1.04–1.13), 2–3 times a week (OR = 1.22, 95% CI = 1.17–1.27), or once a week (OR = 1.25, 95% CI = 1.19–1.32) were associated with higher odds of abnormal weight status. For girls, those who participating in VPA 4–6 times a week (OR = 1.11, 95% CI = 1.06–1.16), 2–3 times a week (OR = 1.20, 95% CI = 1.14–1.25), or once a week (OR = 1.17, 95% CI = 1.11–1.23) all have higher odds of abnormal weight status (i.e., overweight or obesity).

**Conclusion:**

This population-based study suggests that infrequent VPA participation is associated with unhealthy weight status in adolescents compared to their physically active counterparts. Additionally, this association remains consistent in both boys and girls.

## 1 Introduction

In recent decades, there has been a continuous rise in the prevalence of obesity among children and adolescents (1, 2). Data sourced from the World Health Organization (WHO) underscores a significant rise in obesity among children and adolescents, with rates surging from 4% in 1975 to 18% in 2016 (3). In this regard, the high prevalence of obesity has raised public concerns, given its position as the sixth most significant risk factor, it plays a substantial role in the collective disease burden (4). Obesity significantly affects the physical and mental health of young individuals, representing a critical risk factor for their overall quality of life. Childhood and adolescent obesity are closely linked to non-communicable diseases such as cardiovascular diseases, type 2 diabetes, and respiratory conditions (5, 6). Moreover, obesity is also associated with behavioral problems, such as low levels of physical activity and physical fitness (7). Additionally, obesity during early life often establishes a lifelong struggle with weight management. Given the challenged in weight loss, obesity in children and adolescents may increase the likelihood of premature mortality and physical morbidity persisting into adulthood (8). Moreover, obesity in childhood and adolescence is associated with psychological risk factors, including low self-esteem, depression, and anxiety, which can lead to increased adult mortality and morbidity (9). For instance, obese adolescents often face peer victimization, resulting in higher rates of low self-esteem and increased depression compared to their normal-weight peers (10).

It has been known that regular physical activity participation has benefits for healthy weight status in children and adolescents (11–13). Furthermore, a cross-sectional study was conducted to assess if the intensity of physical activity moderates the link between physical activity and weight status among school-aged children (14, 15). For example, Parikh and Stratton (15) conducted a qualitative review and found that higher intensity of PA was the most significant predictor of weight status among children and adolescents. Moreover, a cross-sectional investigation was carried out to examine whether the intensity of physical activity moderates the relationship between physical activity and the risk of overweight or obesity in children (14). The results suggested that lower level of VPA was associated with enhanced odds of overweight and adiposity (14). Therefore, there is a need to examine the relationship between VPA and weight status in children and adolescents. In this regard, some qualitative reviews were conducted and found associations between VPA and weight status in this population (16, 17). For instance, Gralla et al. (17) conducted a narrative review and found that vigorous physical activity (VPA) was more strongly related to body composition in children and adolescents. A recent systematic review and meta-analysis synthesized the correlation between device-measured vigorous physical activity (VPA) and health outcomes, indicating an inverse relationship between VPA and overall adiposity [*r* = −0.09, 95% confidence interval (CI) = −0.15 to −0.03], as well as overweight/obesity (*r* = −0.20, 95%CI = −0.38 to −0.03) in children and adolescents (18). Of note, Gralla et al. (17) also found that some included studies reported insignificant associations between VPA and weight status in children and adolescents. For these reasons, population-based surveys are needed to investigate this relationship among children and adolescents, especially for potential confounding factors.

More importantly, dietary patterns have been recognized as an important factor affecting weight status in children and adolescents. For instance, one review that quantitatively synthesized the data suggested an association between diet and overweight/obesity. It showed that higher intake of sugar-sweetened beverages, fast food, meat and refined grains were associated with increased risk of overweight/obesity among adolescents (19). Moreover, there is also a association between dietary patterns and VPA. For example, Durksen et al. (20) found VPA was associated with healthier dietary patterns in adolescents. Nevertheless, previous studies did not examine diet when investigating the association between VPA and weight status in adolescents may make the results less credible. As an example, one international cross-sectional study involved 199,502 adolescents indicating no association between VPA and body mass index (21). The originality of this study is that it uses a population-based sample to explore the association between VPA and weight status in adolescents, it takes dietary patterns into account. Meanwhile, our study includes data from 45 countries and regions in Europe, Central Asia, and North America, and is based on a large-scale study of the association between vigorous physical activity and weight status. The results of this study can inform the results of adolescent health interventions in an international context.

## 2 Methods

### 2.1 Study design

The 2017/2018 Health Behavior in School-aged Children survey (HBSC) was a collaborative research initiative overseen by the World Health Organization. This cross-sectional study focused on individuals aged 11, 13, and 15, with an average deviation of 0.5 years. Employing a systematic multistage stratified cluster randomized sampling method in each participating country, the HBSC survey aimed for a nationally representative sample. Approximately 1,500 students from each of the specified age groups were chosen in every HBSC country or region. The 2017/2018 survey was conducted in more than 40 countries in Europe, Central Asia, and North America,^1^ across 45 countries and regions with data collected from 24,051 students (22). All the participants in the HBSC survey provided the written consent. The study procedures received ethical approval from the institutional ethics committees in every participating country (22). In total, 227,441 children and adolescents participated in the 2017/2018 survey.

### 2.2 Measures

Participants’ frequency of vigorous physical activity was assessed with the following question: “Outside school hours: how many often do you usually participated in physical activity in your free time that you get out of breath or sweat?” Response options included “Every day,” “4–6 times a week,” “2–3 times a week,” “Once a week,” “Once a month,” “Less than once a month,” “Never.”

Participants were asking about the frequency of consuming breakfast on workdays and weekends, as well as the intake of fruits, vegetables, sweets, soft drinks, meal, smoking, and alcohol. Participants were required to answer the following questions. (1) “How often do you usually have breakfast (more than a glass of milk or juice)?” Response options ranged from “Never” to “5 days” for workdays and “Never” to “Both days” for weekends. (2) “How many times a week do you usually eat fruits, vegetables and sweets or drink soft drinks?” Response options included “Never,” “Less once a week,” “Once a week,” “2–4 days a week,” “5–6 days a week,” “Once daily,” “More than once daily.” (3) “How often do you and your family usually have meals together?.” Responses were “Every day,” “Most days,” “About once a week,” “Less often” and “Never.” (4) “During the past 30 days, how many days (if any) have you smoked cigarettes or drunk alcohol?.” Responses included “Never,” “1–2 days,” “3–5 days,” “6–9 days,” “10–19 days,” “20—29 days” and “30 days (or more).”

Weight status, as indicated by Body Mass Index (BMI), was computed using the formula (kg/m^2^) based on self-reported body weight (“How much do you weigh without clothes?”) and height (“How tall are you without shoes?”). The WHO gender-specific BMI for age growth charts were used to categorize participants’ weight status (23). Children and adolescents falling above the 97th percentile was considered as obese, while those falling between 85th and 97th percentile was classified as overweight (23).

### 2.3 Statistical analysis

All analyses were conducted using IBM SPSS version 28.0. The descriptive characteristics of participants were reported as percentage with standard error and a 95% confidence interval. Ordered logistic regression models were employed to examine the association between vigorous physical activity and weight status across the overall sample, as well as separately for boys and girls. Participating in VPA every day was set as the reference group. All models were adjusted for age category, dietary patterns, and relative family affluence category (except for sex in the overall sample). The statistical significance level was set as *p* < 0.05 in this study.

## 3 Results

Table 1 presents the descriptive characteristics of the sample. 33.8% of participants were 11 years old, 34.5% were 13 years old and 31.8%were 15 years old, with an average age of 13.49 years. The sample comprised 49.3% males and 50.7% females. The mean BMI of the participants was 19.53, range of 11.02–44.91. Regarding weight status, 14.2% were classified as underweight, 69.9% fell within the normal weight range, 13.0% were considered overweight, and 2.9% were classified as obese. The majority of participants engaged in VPA for 2–3 times a week (30.0%), followed by 4–6 times a week (23.6%) and daily (17.4%). A smaller proportion participated in VPA for once a week (13.5%), and less than once a month (4.3%) or not at all (6.8%). On weekdays, 57.9% had breakfast 5 days a week, while 17.9% never had breakfast. During the weekend, 76.4% had breakfast on both days, 15.1% on 1 day, and 8.5% never.

**Table 1:** The descriptive characteristics of the sample.

Variables	Frequent (%)	95%CI	
Sex			
Male	49.3%	49.1%	49.5%
Female	50.7%	50.5%	50.9%
Age			
11 years	33.8%	33.9%	33.9%
13 years	34.5%	34.3%	34.7%
15 years	31.8%	31.6%	31.9%
Weight status			
Thinness	14.2%	14.1%	14.4%
Normal weight	69.9%	69.7%	70.1%
Overweight	13.0%	12.8%	13.1%
Obesity	2.9%	2.8%	3.0%
VPA			
Every day	17.4%	17.2%	17.6%
4–6 times a week	23.6%	23.4%	23.8%
2–3 times	30.0%	29.8%	30.1%
Once a week	13.5%	13.4%	13.7%
Once a month	4.4%	4.3%	4.5%
Less than once a month	4.3%	4.2%	4.4%
Never	6.8%	6.7%	6.9%
Physact60	4.057667	4.05	4.07
Breakfast (weekdays)			
Never	17.9%	17.7%	18.0%
1 day	5.4%	5.3%	5.5%
2 days	5.9%	5.8%	6.0%
3 days	7.1%	7.0%	7.2%
4 days	5.9%	5.8%	6.0%
5 days	57.9%	57.7%	58.1%
Breakfast (weekend)			
Never	8.5%	8.3%	8.6%
1 day	15.1%	14.9%	15.2%
Both days	76.4%	76.3%	76.6%
Fruits			
Never	3.1%	3.1%	3.2%
Less once a week	6.3%	6.2%	6.4%
Once a week	9.4%	9.3%	9.5%
2–4 days a week	25.2%	25.0%	25.4%
5–6 days a week	15.7%	15.5%	15.8%
Once daily	17.6%	17.5%	17.8%
More than once daily	22.6%	22.4%	22.8%
Vegetables			
Never	4.8%	4.7%	4.8%
Less once a week	6.1%	6.0%	6.2%
Once a week	9.5%	9.3%	9.6%
2–4 days a week	22.5%	22.3%	22.7%
5–6 days a week	18.5%	18.4%	18.7%
Once daily	19.4%	19.2%	19.5%
More than once daily	19.3%	19.1%	19.4%
Sweets			
Never	4.1%	4.1%	4.2%
Less once a week	12.4%	12.3%	12.5%
Once a week	18.1%	17.9%	18.2%
2–4 days a week	27.3%	27.1%	27.4%
5–6 days a week	12.9%	12.8%	13.1%
Once daily	12.7%	12.6%	12.8%
More than once daily	12.5%	12.4%	12.6%
Soft drinks			
Never	14.0%	13.9%	14.2%
Less once a week	23.9%	23.7%	24.1%
Once a week	18.9%	18.8%	19.1%
2–4 days a week	19.1%	19.0%	19.3%
5–6 days a week	8.1%	8.0%	8.2%
Once daily	6.8%	6.7%	6.9%
More than once daily	9.1%	9.0%	9.2%
Meal			
Every day	49.9%	49.7%	50.1%
Most days	32.0%	31.8%	32.2%
About once a week	9.0%	8.9%	9.1%
Less often	6.6%	6.5%	6.7%
Never	2.5%	2.5%	2.6%
Smoking			
Never	93.1%	93.0%	93.2%
1–2 days	2.3%	2.3%	2.4%
3–5 days	0.9%	0.9%	1.0%
6–9 days	0.6%	0.6%	0.7%
10–19 days	0.6%	0.6%	0.7%
20–29 days	0.6%	0.5%	0.6%
30 days (or more)	1.8%	1.7%	1.8%
Alcohol			
Never	81.2%	81.0%	81.3%
1–2 days	11.5%	11.4%	11.7%
3–5 days	3.6%	3.6%	3.7%
6–9 days	1.7%	1.6%	1.7%
10–19 days	0.8%	0.8%	0.9%
20–29 days	0.3%	0.3%	0.3%
30 days (or more)	0.9%	0.8%	0.9%
Relative family affluence categorical			
Lowest	19.8%	19.6%	19.9%
Medium	61.4%	61.2%	61.6%
Highest	18.8%	18.6%	19.0%

Regarding dietary habits, 22.6% of participants consumed fruit more than once daily, while 9.4% consumed fruit once a week, and 6.3% less than once a week. For vegetable consumption, 38.6% consumed vegetables once a day or more, while a smaller proportion consumed vegetables less frequently. In terms of sweet consumption, 25.2% consumed sweets once daily or more, and 34.6% consumed sweets once a week or less. Additionally, only 15.9% consumed soft drinks once daily or more, with the majority consuming them once a week or less. Almost half of the participants consumed meals daily (49.9%), and the majority did not smoke (93.1%) or drink alcohol regularly (81.2%). Regarding relative family affluence categorical, 61.4% were at a medium level, with smaller proportions in the lowest 20% (19.8%) or highest 20% (18.8%).

Table 2 illustrates the association between vigorous physical activity (VPA) frequency and weight status across the entire sample. It reveals that, compared to daily VPA, less frequency is linked to higher odds of obesity. For example, those who participated in VPA for 4–6 times a week (OR = 1.10, 95% CI = 1.06–1.13), 2–3 times a week (OR = 1.21, 95% CI = 1.17–1.25), or once a week (OR = 1.21, 95% CI = 1.16–1.25) all have higher odds of abnormal weight status. In terms of covariates, females (OR = 0.62, 95% CI = 0.61–0.64) are less likely to have abnormal weight status compared to males. Older participants (OR = 1.07, 95% CI = 1.06–1.08) are more likely to have abnormal weight status. Breakfast consumption during weekdays (OR = 0.94, 95% CI = 0.93–0.94) and weekends (OR = 0.93, 95% CI = 0.91–0.95) is associated with reduced odds of abnormal weight status. Higher fruit consumption (OR = 1.01, 95% CI = 1.00–1.02) is linked to higher odds of abnormal weight status, while sweet consumption (OR = 0.91, 95% CI = 0.90–0.91) is associated with lower odds. Moreover, regular meal consumption (OR = 1.08, 95% CI = 1.07–1.09), smoking (OR = 1.02, 95% CI = 1.01–1.03), and alcohol consumption (OR = 1.06, 95% CI = 1.05–1.07) are all related to higher odds of abnormal weight status. Lastly, relative family affluence categorical (OR = 0.87, 95% CI = 0.85–0.89) is associated with lower odds of abnormal weight status.

**Table 2:** The association between VPA and weight status in overall sample.

IOTF4	Odds ratio	Std. err.	*t*	*P* > *t*	[95% conf. interval]	
VPA						
Every day	REF					
4–6 times a week	1.10	0.02	5.78	<0.001	1.06	1.13
2–3 times a week	1.21	0.02	12.47	<0.001	1.17	1.25
Once a week	1.21	0.02	10	<0.001	1.16	1.25
Once a month	1.20	0.03	6.55	<0.001	1.14	1.27
Less than once a month	1.31	0.04	9.6	<0.001	1.24	1.39
Never	1.17	0.03	6.69	<0.001	1.11	1.22

All models were controlled for sex, age, relative family affluence categorical, breakfast on workdays and weekend, intake of fruits, vegetables, sweets, soft drinks, meal, smoking, and alcohol; Bold numbers: statistically significant at *p* < 0.05.

Table 3 shows the association between VPA frequency and weight status in boys. It reveals that, compared to daily VPA, less frequency is linked to higher odds of abnormal weight status. For example, those who participated in VPA for 4–6 times a week (OR = 1.09, 95% CI = 1.04–1.13), 2–3 times a week (OR = 1.22, 95% CI = 1.17–1.27), or once a week (OR = 1.25, 95% CI = 1.19–1.32) all have higher odds of abnormal weight status. In terms of covariates, older participants (OR = 1.08, 95% CI = 1.06–1.10) are more likely to have abnormal weight status. Breakfast consumption during weekdays (OR = 0.94, 95% CI = 0.93–0.94) and weekends (OR = 0.93, 95% CI = 0.91–0.95) is associated with reduced odds of abnormal weight status. Higher fruit consumption (OR = 1.01, 95% CI = 1.00–1.02), regular meal consumption (OR = 1.07, 95% CI = 1.06–1.09), smoking (OR = 1.02, 95% CI = 1.01–1.04), and alcohol consumption (OR = 1.06, 95% CI = 1.05–1.08) are all related to higher odds of abnormal weight status. Conversely, sweet consumption (OR = 0.91, 95% CI = 0.90–0.92) and relative family affluence categorical (OR = 0.89, 95% CI = 0.87–0.91) are associated with lower odds of abnormal weight status.

**Table 3:** The association between VPA and weight status in boys.

IOTF4	Odds ratio	Std. err.	*t*	*P* > *t*	[95% conf. interval]	
VPA						
Every day	REF					
4–6 times a week	1.09	0.0222658	4.08	<0.001	1.04	1.13
2–3 times a week	1.22	0.0248058	9.83	<0.001	1.17	1.27
Once a week	1.25	0.0339764	8.36	<0.001	1.19	1.32
Once a month	1.18	0.0510864	3.9	<0.001	1.09	1.29
Less than once a month	1.39	0.0592826	7.82	<0.001	1.28	1.52
Never	1.21	0.0414515	5.57	<0.001	1.13	1.29

All models were controlled for sex, age, relative family affluence categorical, breakfast on workdays and weekend, intake of fruits, vegetables, sweets, soft drinks, meal, smoking, and alcohol; Bold numbers: statistically significant at *P* < 0.05.

Table 4 presents the association between VPA frequency and weight status in girls. It reveals that, compared to daily VPA, less frequency is associated with higher odds of abnormal weight status. For instance, those who participated in VPA for 4–6 times a week (OR = 1.11, 95% CI = 1.06–1.16), 2–3 times a week (OR = 1.20, 95% CI = 1.14–1.25), or once a week (OR = 1.17, 95% CI = 1.11–1.23) all have higher odds of abnormal weight status. In terms of covariates, older participants (OR = 1.07, 95% CI = 1.05–1.09) are more likely to have abnormal weight status. Breakfast consumption during weekdays (OR = 0.94, 95% CI = 0.93–0.94) and weekends (OR = 0.93, 95% CI = 0.91–0.96) is associated with reduced odds of abnormal weight status.

**Table 4:** The association between VPA and weight status in girls.

IOTF4	Odds ratio	Std. err.	*t*	*P* > *t*	[95%conf. interval]	
VPA						
Every day	REF					
4–6 times a week	1.11	0.0274137	4.19	<0.001	1.06	1.16
2–3 times a week	1.20	0.0281858	7.62	<0.001	1.14	1.25
Once a week	1.17	0.0314526	5.86	<0.001	1.11	1.23
Once a month	1.20	0.0448791	4.98	<0.001	1.12	1.30
Less than once a month	1.25	0.0482306	5.83	<0.001	1.16	1.35
Never	1.13	0.0356745	3.81	<0.001	1.06	1.20

All models were controlled for sex, age, relative family affluence categorical, breakfast on workdays and weekend, intake of fruits, vegetables, sweets, soft drinks, meal, smoking, and alcohol; Bold numbers: statistically significant at *P* < 0.05.

Higher fruit consumption (OR = 1.01, 95% CI = 1.00–1.02), regular meal consumption (OR = 1.08, 95% CI = 1.07–1.10), smoking (OR = 1.01, 95% CI = 1.00–1.03), and alcohol consumption (OR = 1.06, 95% CI = 1.04–1.08) are all related to higher odds of abnormal weight status. Conversely, sweet consumption (OR = 0.91, 95% CI = 0.90–0.92) and relative family affluence categorical (OR = 0.85, 95% CI = 0.83–0.87) are associated with lower odds of abnormal weight status.

## 4 Discussion

This study based on a population sample examined the association between participation in vigorous physical activity (VPA) and weight status among adolescents. The findings suggested that, compared to adolescents who participated in VPA more frequently, those who engaged in less frequent VPA are more likely to have higher odds of unhealthy weight status. Our findings are consistent with several previous studies (24, 25). For example, Gutin et al. (26) investigated 421 high school students and found that lower amount of VPA participation was associated with high body fat [*β* = −4.19, SE (standard error) = 1.02, *p* = 0.001]. Another cross-sectional study involved 409 children using accelerometers found a positive association between VPA participation and normal weight status in boys [OR (odd ratios) = 1.13, 95%CI = 1.03 to 0.23, *p* = 0.01] and girls (OR = 1.13, 95%CI = 1.02–1.25, *p* = 0.03) (24). However, an international cross-sectional study of 199,502 adolescents found that no significant difference in BMI between those who did not participate in VPA and those who participated frequently (BMI: +0.01 kg/m^2^, 95%CI = −0.03–0.05) (21). Additionally, participants who infrequently engaged in VPA (BMI: +0.19, 95%CI = 0.15–0.23) had higher BMI values when compared to did not participated in VPA (21). As discussed in the previous study, the slight higher BMI values may be attributed to the greater muscle mass gained from participating in VPA when compared to their physically inactive counterparts (21). Notably, the previous study did not include potential covariates, which could serve as an additional explanation for the insignificant association between VPA participation and BMI among adolescents. It is well-established that factors beyond physical activity, such as age, gender, family affluence and diet, are all associated with weight status in adolescents (27). Therefore, incorporating potential covariates may enhance our understanding of the relationship between VPA and weight status in this population.

Moreover, as an important factor affecting weight status in children and adolescents, dietary patterns may provide another explanation for the insignificant association observed in the previous study (19). Individuals who engage in higher doses of physical activity experience a dietary caloric compensation, characterized by an increased intake of calorie dense, nutrient deficient foods (28). Diet, as the primary way of energy intake for adolescents, also has a direct contribution to obesity. Increased dietary calories in adolescents increase the possibility of obesity (29). In addition, Durksen et al. (20) found that VPA was associated with healthier dietary patterns, and physically active adolescents tend to consume less unhealthy food when compared to their less active counterparts. All of this evidence suggests that that dietary patterns may significantly influence the association between VPA and weights status. Therefore, when addressing adolescents’ weight status, it is essential to consider the interplay between VPA, weights status and diet simultaneously. Our findings underscore the importance of identifying more potential factors that may influence the association between VPA and weight status in adolescents.

Our findings indicated that infrequent participation in VPA was consistently associated with higher BMI in both boys and girls. Previous studies have also highlighted sex differences in the relationship between VPA and weight status. For instance, a longitudinal study involving 462 youth suggested that girls, whether with or without obesity, exhibit lower amount VPA compared to boys (30). A systematic review has qualitatively synthesized the relationship between objectively measured VPA and weight status, suggesting a potential differential association between VPA and weight status for boys and girls (17). However, to better understand the moderating effects of sex, more robust evidence is warranted. Notably, a substantial disagreement exists between subjective (e.g., questionnaire-based) and objective (e.g., accelerometer-based) measures when collecting VPA data among adolescents (31). Therefore, conducting population-based surveys with reliable and valid assessment is essential to enhance our comprehension of the relationship between VPA and weight status in adolescents.

The significant association between physical activity and weight status can be explained by energy expenditure. Compared to low intensity of physical activity, vigorous physical activity can enhance adolescents’ energy expenditure, potentially achieving weight loss. Moreover, VPA plays a crucial role in regulating various hormones and myokines related to body composition, which may impact energy balance and appetite control, potentially alleviating hunger in obese individuals (32, 33). Conversely, VPA could enhance fat oxidation, particularly in high-fat diets (32, 33). Physiologically, VPA may facilitate greater fat utilization, possibly linked to quantitative and qualitative mitochondrial adaptations. Significantly, this encompasses notable enhancements in skeletal muscle mitochondrial volume and density, along with inherent fatty acid oxidation within the mitochondria (34). Furthermore, VPA elicits a heightened catecholamine response in individuals, with greater physical activity intensity corresponding to higher catecholamines concentration (35). Altered catecholamine responses influence the sensitivity of alpha and beta adrenergic receptors in adipose tissue, leading to reduced lipolysis and increased fat storage. Catecholamines play a crucial role in driving lipolysis both during and after physical activity, thereby impacting fat mobilization and storage (36).

## 5 Strengths and limitations

One notable strength of this study lies in its internationally representative sample of adolescents from 45 countries, encompassing a wide range of socioeconomic background. The adoption of a standardized methodological protocol for data collection across these diverse countries serves to mitigate potential measurement biases, particularly when conducting an international survey. Although a large number of studies have confirmed the negative association between VPA and adolescent obesity, others have found no significant association (37). This issue may be a result of the complexity of energy balance, and adolescent obesity is not only influenced by physical activity but also by other confounding factors such as dietary intake, hormonal regulation and genetic variation (38). There are also limitations to this study. Firstly, although it has controlled for dietary intake such as breakfast, fruits and vegetables and smoking other health behaviors, may not have fully covered all the factors that influence obesity. For example, ethnic origin of the participants and the level of sexual maturity in adolescents may be related to weight status but was not controlled for due to study conditions. Secondly, this study is cross-sectional design, which limits the ability of this study to draw causal conclusions. Thirdly, this study used BMI to define obesity in children and adolescents, this may have errors, for example children with the same BMI may have different muscle mass and fat mass. Fourthly, this study was based on self-report and there may be some reporting bias, adolescents may have underestimated their weight and overestimated their vigorous physical activity. Fifthly, the VPA for this study used data from outdoor sessions based on breathlessness and sweating. This may deviate from the actual performance of VPA. On the one hand, there are many factors that may influence the performance of outdoor vigorous intensity activities in different regions. On the other hand, short, intermittent periods of intense activity may not be included.

## 6 Conclusion

This population-based study suggested that infrequent VPA participation was associated with unhealthy weight status in adolescents compared to their physically active counterparts. Additionally, this association was consistent in both boys and girls. This emphasizes the importance of VPA in maintaining healthy weight status. In future, more population-based surveys are needed to identify potential confounding factors, which can provide a better understanding of this relationship in young individuals.

## Data availability statement

Publicly available datasets were analyzed in this study. This data can be found at: https://hbsc.org/data/.

## Ethics statement

Written informed consent was obtained from the individual(s), and minor(s)’ legal guardian/next of kin, for the publication of any potentially identifiable images or data included in this article.

## Author contributions

WY: Conceptualization, Data curation, Methodology, Writing – original draft, Writing – review & editing. SX: Data curation, Formal analysis, Writing – review & editing. XD: Data curation, Writing – review & editing. HF: Methodology, Software, Writing – review & editing.

## Footnotes

^1^ www.hbsc.org
